# Identification of residues in ABCG2 affecting protein trafficking and drug transport, using co-evolutionary analysis of ABCG sequences

**DOI:** 10.1042/BSR20150150

**Published:** 2015-08-20

**Authors:** Ameena J. Haider, Megan H. Cox, Natalie Jones, Alice J. Goode, Katherine S. Bridge, Kelvin Wong, Deborah Briggs, Ian D. Kerr

**Affiliations:** * School of Life Sciences, University of Nottingham, Queens Medical Centre, Nottingham NG7 2UH, U.K.

**Keywords:** ATP-binding cassette (ABC) transporter, drug binding, glycosylation, trafficking, transmembrane helix

## Abstract

ABCG2 is an ABC (ATP-binding cassette) transporter with a physiological role in urate transport in the kidney and is also implicated in multi-drug efflux from a number of organs in the body. The trafficking of the protein and the mechanism by which it recognizes and transports diverse drugs are important areas of research. In the current study, we have made a series of single amino acid mutations in ABCG2 on the basis of sequence analysis. Mutant isoforms were characterized for cell surface expression and function. One mutant (I573A) showed disrupted glycosylation and reduced trafficking kinetics. In contrast with many ABC transporter folding mutations which appear to be ‘rescued’ by chemical chaperones or low temperature incubation, the I573A mutation was not enriched at the cell surface by either treatment, with the majority of the protein being retained in the endoplasmic reticulum (ER). Two other mutations (P485A and M549A) showed distinct effects on transport of ABCG2 substrates reinforcing the role of TM helix 3 in drug recognition and transport and indicating the presence of intracellular coupling regions in ABCG2.

## Abstract (precis)

Determining how efflux pumps function is important to understanding their role in drug resistance. We have identified amino acids in a human drug efflux pump that affect interaction with substrate and protein targeting.

## INTRODUCTION

ABCG2 is one of the five members of the G-subfamily of human ATP-binding cassette (ABC) transporters. The protein is localized to numerous epithelial tissues within the body, including much of the gastrointestinal tract. It is also found in the kidneys, the placenta and within the endothelium of the vessels of the blood–brain barrier [1]. It is one of the three human ABC transporters that are well characterized as multi-drug pumps, i.e. having a broad specificity for substrates that encompasses molecules with different chemical structures and physico-chemical properties [2]. This broad substrate specificity, together with the tissue distribution data, implicates ABCG2 as having a protective role by exporting xenobiotic compounds from organs. As such it is a major factor in the pharmacokinetics of prescribed drugs including statins [3] and fluoroquinolone antibiotics [4]. Moreover, ABCG2 is able to transport many anti-cancer and anti-neoplastic agents and up-regulated expression of ABCG2 is observed in some forms of drug-resistant cancer [5]. A further physiological role has come from the demonstration that the most common polymorphism of ABCG2 (rs2231142) is associated with gout and that ABCG2 is a transporter of urate in the kidneys [6,7].

ABCG2 (in common with other members of the human ABCG sub-family) has two topological features that are distinct from many other eukaryotic ABC transporters. Firstly, it is a ‘half transporter’, being composed of only a single transmembrane domain (TMD) and a single nucleotide-binding domain (NBD) in the polypeptide chain. This has led to the assertion that ABCG2 must at least homo-dimerize in order to function, although the exact oligomeric state is unknown [8–10]. Another impediment to our understanding of ABCG2 is that its domains are in the reverse order compared with the majority of ABC proteins. In other words, the NBD is preceded by the TMD, whereas the typical configuration has the TMD N-terminal to the NBD. This combination of features means that it is impossible to infer structure: function information by comparison with other, better characterized human multi-drug pumps such as ABCB1/P-glycoprotein [11]. Notwithstanding this, a number of homology models for ABCG2 exist but these may not be an ideal structural framework to explain data or make new predictions about important residues and regions [12,13].

To date, ABCG2 substrate interaction has been characterized by radioligand-binding analysis and has been identified to have a complex network of drug interactions whose affinities are altered by the interaction of NBDs with ATP [14,15]. The spatial localization of drug interaction site(s) is unknown, with only one well-characterized example of a residue known to impact upon drug selectivity. Residue 482 in transmembrane helix (TM) 3 of the TMD is an arginine in the wild-type (WT) sequence, but a drug selected cell line expressing a mutant version (where the arginine is replaced by glycine; R482G) shows a broader resistance profile including doxorubicin and rhodamine 123 in its substrates [16–18]. Structurally, there are two EM studies of ABCG2. Although these are limited in their resolution, they are able to confirm that the transporter can form stable homo-oligomers and undergoes conformational changes in response to drug binding [10,19].

Our group is interested in understanding the structure and function of ABCG2. Due to the limitations of using homology models of ABCG2 based upon other ABC exporters, we have adopted a sequence analysis based approach to identify residues in ABCG2 for mutational analysis and characterization. Using co-evolution analysis (i.e. the correlated changes of amino acids at spatially distinct positions in the primary sequence), we have mutated several amino acids in the NBD and TMD of ABCG2. Analysis of these mutated isoforms enabled us to identify residues that contribute to drug binding or allosteric communication and to trafficking of ABCG2 to the membrane.

## MATERIALS AND METHODS

### Molecular biology

All molecular biology enzymes were from either New England Biolabs (NEB) or Promega. The mutant isoforms were generated in an N-terminally His_12_ tagged version of WT ABCG2 in pcDNA3.1(+). The mutants were constructed using Quikchange II technology (Stratagene) employing complementary primers (Sigma) each encoding the mutation of interest. Primers also included a further mutation, synonymous in terms of translation, to alter the restriction digest profile if the introduction of the mutant did not accomplish this. All primers are listed in Supplementary Table S1. Following PCR, competent DH5α cells were transformed with the reaction products and colonies screened for plasmids showing the modified restriction digest profile. Superfolder GFP (sfGFP)-tagged variants of ABCG2 were constructed by sub-cloning the ABCG2 coding region into a pre-existing p3.1zeo_sfGFP-ABCG2 vector (Wong and Kerr, unpublished). All plasmids were sequenced across the entirety of the ABCG2 cDNA to ensure the fidelity of mutagenesis and sub-cloning.

### Cell culture

Human embryonic kidney (HEK) 293T cells were maintained in Dulbecco's Modified Eagle Media (DMEM; Sigma) supplemented with 10% foetal calf serum (FCS; Gibco) and 100 units/ml of penicillin/streptomycin (Invitrogen) at 37°C in a humidified 5% CO_2_ incubator. Cells were transfected using polyethyleneimine (linear PEI, Polysciences) [20]. For six-well dishes cells were plated at 250000–300000 per well, 24 h prior to transfection. Three hours prior to transfection, the media was replaced by a 5% serum containing DMEM. Plasmid DNA (2–4 μg) was mixed with PEI at a nitrogen–phosphorus molar ratio of 15:1 [20] and was added to the cells within a few minutes of complex formation. One day later, the media was replaced by DMEM–10% FCS. For flow cytometry transfection was scaled up to 10-cm dishes. Stable HEK293T cell lines expressing sfGFP–ABCG2 were obtained by supplementing media 24 h post transfection with zeocin (Sigma; 200 μg/ml) for 7–14 days until cell death was complete in untransfected cells and when zeocin-resistant colonies had developed in plasmid transfected cells. Subsequently stable, variable expression level cell lines were passaged in media containing 40–50 μg/ml zeocin.

### SDS/PAGE and immunoblotting

Cells were harvested by repeat pipetting and then centrifuged (14000 ***g***, 1 min), washed in ice-cold PBS and re-pelleted as above. Cells were resuspended in ice-cold PBS supplemented with protease inhibitors (Complete EDTA-free Protease Inhibitor, Roche) and lysis was performed by 3×10 s bursts with a chilled probe sonicator (Microsonix). Protein concentration was determined using a modified Lowry protein assay (Biorad) and cell lysates (30 μg of protein) were resolved by SDS/PAGE [8%–10% (w/v) acrylamide]. Following transfer on to nitrocellulose membrane, ABCG2 was detected using BXP-21 (Merck Biosciences) at a 1:500–1:2000 dilution in PBS/5% (w/v) skimmed milk powder/0.1% (v/v) Tween, followed by a horseradish peroxidase-conjugated rabbit anti-mouse secondary antibody (1:2000; DAKO). Chemiluminescence was detected using Supersignal West Pico (Pierce). Parallel gels were loaded and stained with Coomassie Brilliant Blue to ensure equal protein loading.

### Deglycosylation of ABCG2

Cell lysates (typically 30 μg of protein) were combined with 10× glycoprotein denaturing buffer (5% SDS, 0.4 M DTT) and H_2_O to make a 10 μl of reaction volume. The samples were then denatured by heating at 100°C for 10 min. Following this, the total reaction volume was adjusted to 20 μl by adding 10× G7 reaction buffer [0.5 M sodium phosphate (pH 7.5 at 25°C)], 10% NP40, H_2_O and 1–2 μl of peptide–N-glycosidase F (PNGaseF) and incubated at 37°C for 1 h. Samples were then resolved by SDS/PAGE.

### Expression and functional analysis by flow cytometry

HEK293T cells were analysed 36–40 hrs post-transfection by flow cytometry. Cells were washed several times in ice-cold sterile PBS and pelleted by centrifugation at 150 ***g*** for 5 min at 4°C. Pellets were resuspended in flow cytometry buffer [1% (v/v) BSA in phenol red-free DMEM] and aliquotted into flow cytometry tubes as 100 μl of aliquots at a cell density of 1–2×10^6^ cells per ml. For cell surface expression, cells were incubated with 5D3-PE (anti-ABCG2 antibody 5D3 conjugated to phycoerythrin; R&D systems) at a 1:100 dilution. Parallel cell aliquots were incubated with isotype control antibodies (IgG-PE; 1:100 dilution; MACS). For mitoxantrone (MX) accumulation cells were incubated in the presence of 5 μM MX (Sigma) in the presence or absence of fumitremorgin C (FTC; 10 μM; Sigma). Parallel vehicle controls contained the maximum solvent concentration [DMSO, <0.5% (v/v)] to ensure that cell viability was unaffected by the solvent. Following incubation at 37°C with occasional agitation for 30 min, cells were centrifuged at 300 ***g*** for 1–3 min at 4°C. The pellets were then washed twice with ice-cold flow cytometry buffer and finally resuspended in 300–400 μl of buffer, prior to analysis using a Beckman-Coulter XL-MCL Flow cytometer. PE fluorescence was determined using excitation at 546 nm and emission at 578 nm and MX fluorescence measured using excitation at 635 nm and emission at 670 nm. Flow cytometry data were analysed using WEASEL v3.1 (The Walter and Eliza Hall Institute of Medical Research).

### Fluorescence microplate transport assay

Black-sided, clear-bottom 96-well plates (Greiner) were incubated for 1 h in 10μg/ml poly-L-lysine (Sigma) before cells were seeded at 40000 cells per well. Plates were incubated overnight at 37°C and 5% CO_2_ before media was replaced with phenol red-free DMEM containing transport substrates [8 μM MX, rhodamine 123 (R123) or pheophorbide A (PhA)] in the presence or absence of 0.5 μM Ko143 (Sigma). Cells were incubated for 1 h at 37°C and were subsequently washed once in PBS. Cells were incubated for a further 1 h at 37°C in phenol red-free DMEM alone, supplemented with Ko143 where required. Cells were washed with ice-cold PBS, before incubation with paraformaldehyde [PFA, 4% (w/v), 15 min] and two final washes with PBS. Cellular fluorescence was determined using a fluorescence plate reader (MDC Flexstation). Fluorescence data were corrected for values obtained from incubations with 1% v/v DMSO, which was the maximum solvent concentration used.

### Fluorescence microscopy and live cell imaging

For live cell imaging, HEK293T cells stably transfected with sfGFP–ABCG2 isoforms were plated on to MatTek glass-bottomed 35-mm dishes at least 24 h prior to imaging and were visualized on a Zeiss LSM 710 (Zeiss) confocal microscope, using a plan-apochromat 63×/1.40 Oil Ph3 M27 objective and argon laser. For immunofluorescence determination of the localization of the I573A isoform, cells were fixed on cover slips with 4% PFA in PBS for 5 min at room temperature before treatment with 50 mM NH_4_Cl for 10 min to quench the free aldehyde groups of the fixative preventing auto-fluorescence [21]. Cells were then washed twice with ice-cold PBS and incubated in 0.5% (w/v) BSA in PBS for 15 min at room temperature to prevent non-specific antibody interaction. Following blocking, cells were incubated for 1 h with anti-calnexin primary antibody (Sigma) prepared at 1:500 dilution in blocking buffer. The primary antibody solution was removed and the cells washed several times with blocking buffer. Cells were then incubated in secondary antibody (donkey anti-goat monoclonal antibody conjugated to AlexaFluor 647 red fluorescent dye; Invitrogen), at a 1:1000 dilution in blocking buffer. Cells were washed several times with blocking buffer and once with PBS, then mounted on to microscope slides with FluoroGel mounting medium (GeneTex), before confocal microscopy as above.

### Bioinformatics analysis

All sequence identification and alignment was performed using standard web-based servers BLAST, ExPASy and ClustalW. Analysis of residues under co-evolutionary selection was performed using the web server (http://coevolution.gersteinlab.org/coevolution/), described in [22].

### Experimental data analysis

All numerical data manipulations were performed using Excel or GraphPad Prism and statistical analyses performed using Prism. All statistical tests are detailed in appropriate figure legends and significance was judged at *P*-values less than 0.05.

## RESULTS

A number of mutations in ABCG2 have been constructed previously to analyse protein function [16,23,24]. We embarked on a directed mutagenesis strategy to identify other amino acids outside of typical conserved ABC transporter regions that are important for function, using co-evolutionary sequence analysis (Supplementary Figure S1; [22]). A residue (i) in a multiple sequence alignment is co-evolving with another residue (j) in that alignment if it can be shown that the evolutionary changes occurring at position i are correlated with amino acid substitutions at position j. This has been shown to be a useful approach to identify pairs of important residues when combined with structural data [25,26]. In the absence of high resolution structural data for ABCG2 and with no compelling homology models [1,11], we used co-evolutionary analysis algorithms to identify residues within the ABCG sub-family that are involved in a ‘network’ of co-evolutionary relationships with multiple other residues. Our reasoning was that these amino acids may be central to the function of ABCG2, be that in coupling of the NBDs to the TMDs or the binding of substrates and inhibitors. Three such co-evolutionary analyses were performed (using a web-based interface) [22] on a multiple sequence alignment of over 100 ABCG family sequences, namely statistical coupling analysis [25], Chi-squared test [27] and explicit likelihood of subset co-variation [28].

This analysis (Supplementary Figure S1; Supplementary Table S2) allowed us to identify eight amino acids (Figure 1) which are frequently involved in co-evolutionary relationships (‘coupled’) with other residues in ABCG2. Notably, all these are coupled to residues in different domains, i.e. the NBD residues identified are coupled to residues in the TMDs and vice versa perhaps suggestive of a role in inter-domain communication. Two of the residues are located in the NBD; Met^131^ which is five amino acids C-terminal from the conserved Q-loop [29] and Ser^195^ which is located five amino acids C-terminal to the signature motif [29]. The other six amino acid mutations are in the TMD and, according to the experimentally determined topology map of Mao and colleagues [30], are located as follows: Lys^453^ in the first turn of TM2, Lys^473^ in the short intracellular loop between TM2 and TM3, Pro^485^ is one turn of α-helix C-terminal to the functionally significant Arg^482^ in TM3, Met^549^ is in the long intracellular loop between TM4 and 5, Trp^564^ is the very intracellular end of TM5 and Ile^573^ is located in TM5. We made individual substitutions of each amino acid to alanine: M131A, S195A, K453A, K473A, P485A, M549A, W564A and I573A. A further three control mutations were analysed, namely a substitution of lysine to alanine in the Walker-A motif (K86A), a substitution of glutamate to glutamine in the Walker-B motif (E211Q) and a substitution of asparagine to glutamine at the site of glycosylation (N596Q). All 11 mutants are displayed schematically in Figure 1.

**Figure 1:**
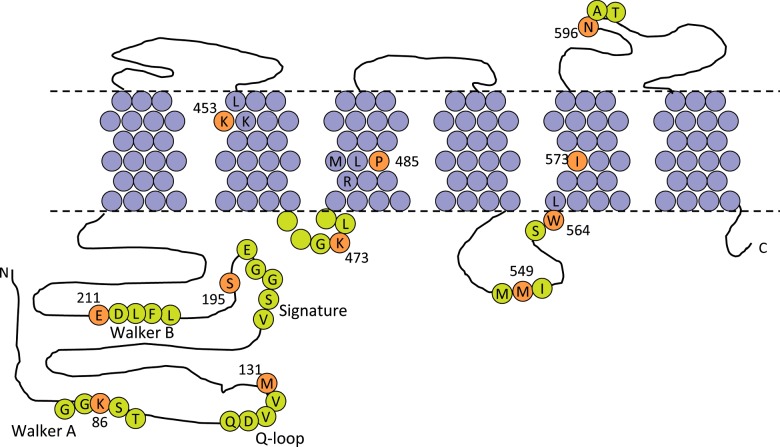
Location of the residues selected for mutation in ABCG2 The transmembrane topology of ABCG2 is represented according to the data of Mao and colleagues [30], with approximate membrane plane indicated by the dashed line. TM residues are shaded purple and extramembraneous residues in lime. Residues for mutation are on an orange background, numbered and indicated using the single-letter amino acid code. Local sequence context and NBD sequence motifs have been shown for ease for reference.

To identify whether the mutants had any dramatic effect on the expression and localization of ABCG2, we transiently transfected plasmid DNA encoding His_12_-ABCG2 isoforms into HEK293T cells and analysed using flow cytometry with antibodies recognizing the extracellular 5D3 epitope. The majority of the mutants were present at the cell surface at comparable levels to the WT (i.e. N-terminally His_12_-tagged ABCG2) protein (e.g. Figures 2B–2D). Analysis of the expression level (given by the ratio 5D3-PE fluorescence labelling compared with IgG–PE fluorescence labelling) of all mutants suggested that, of the novel mutants examined in the present study, only I573A had reduced cell surface expression (Figures 2E and 2F). To ensure that any variability in expression of I573A was not a reflection of the conformational sensitivity of the 5D3 antibody [31], we performed western blotting of whole cell lysates with the BXP-21 antibody which recognizes an intracellular epitope. This confirmed that I573A was expressed at reduced levels compared with other isoforms (Figure 2G; I573A expression is essentially undetectable in this exposure).

**Figure 2:**
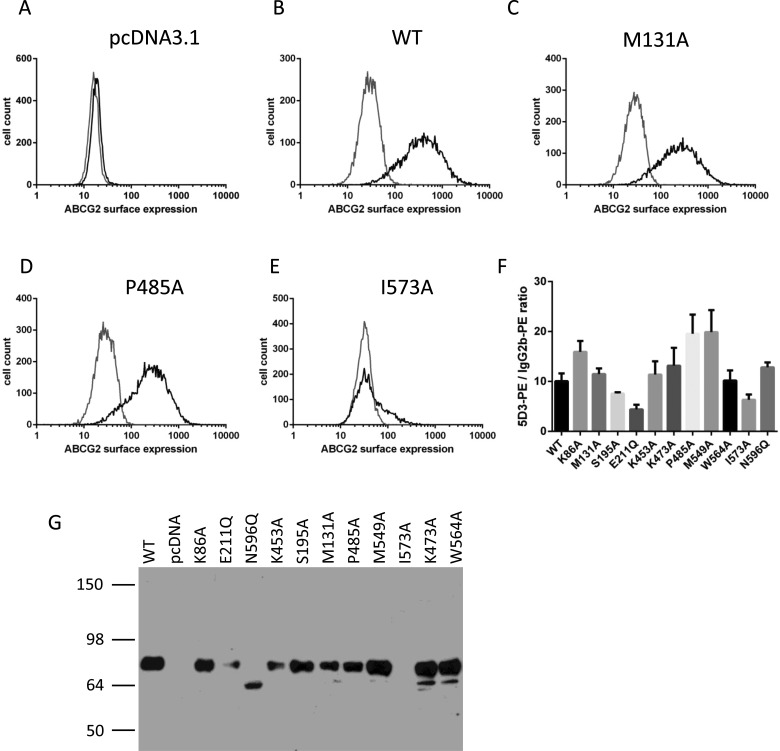
Transient expression of His_12_–ABCG2 and isoforms in HEK293T cells Flow cytometry analysis of cell surface expression 40 h post-transfection (**A–E**). Black lines represent distributions of cellular fluorescence following labelling with PE-conjugated anti-ABCG2 antibody 5D3. Grey lines represent isotype control labelling. (**A–E**) Representative data of the empty vector, WT or named isoforms. The relative surface expression (**F**) was quantified as the ratio of the mean fluorescence values in the presence of 5D3 to the mean fluorescence value in the presence of isotype control antibodies. Mean (± S.E.M.) is shown for at least four independent transfections. (**G**) Western blotting of total ABCG2 expression was determined by resolving 30 μg of whole cell lysate on 10% w/v acrylamide gels and probing with BXP-21 antibody. A representative image of at least four independent experiments is shown.

The failure of one of the isoforms (I573A) to effectively reach the cell surface piqued our interest as membrane trafficking is clearly of cellular and clinical relevance, both for ABCG2 and for other ABC transporters [6,32]. Examination of a time course of protein expression (24 and 48 h post-transfection respectively) confirmed that His_12_I573A was being expressed (Figure 3A), but that the protein appeared to have a reduced molecular mass compared with WT His_12_ABCG2 and a fully functional mutant isoform (His_12_M131A–functional data in Figure 5). Indeed the molecular mass was closer to that of the isoform His_12_N596Q, which cannot be glycosylated due to the removal of the sole ABCG2 N-glycosylation site [33]. Effective glycosylation of membrane proteins is critical in the regulation of their trafficking to the cell surface; so, we further examined the glycosylation of the His_12_I573A isoform 24 and 48 h post-transfection and demonstrated that the slight difference in molecular mass between His_12_I573A and His_12_N596Q is consistent with core glycosylation (Figure 3B). Maturation of the His_12_I573A isoform to a fully glycosylated protein is clearly impaired with the core glycosylated form still in evidence 48 h post transfection (Figure 3B lower panel) compared with other isoforms which are fully glycosylated at 24 h.

**Figure 3:**
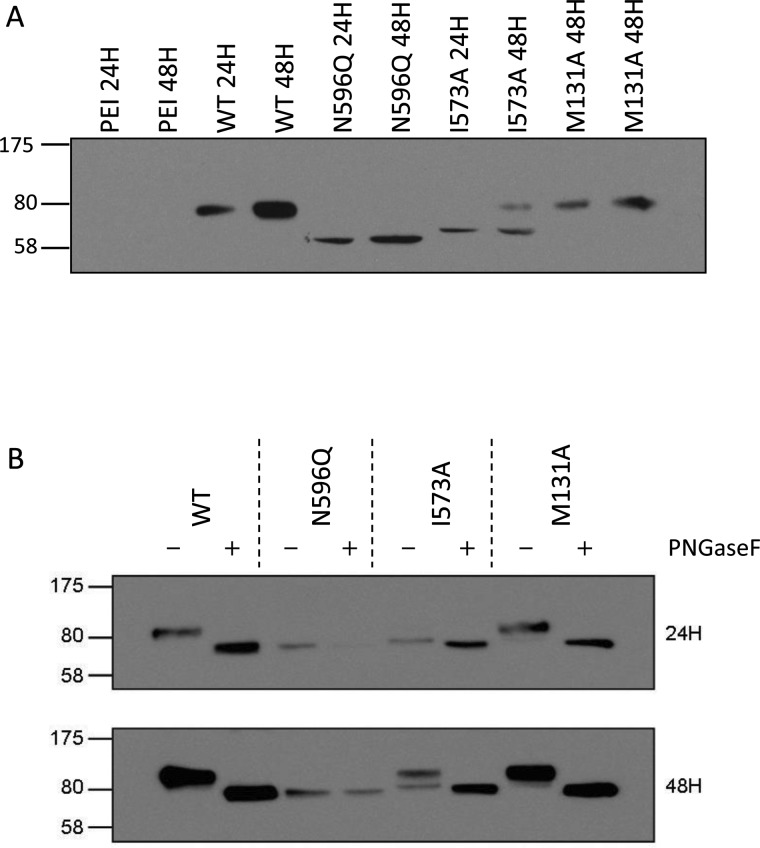
His_12_-I573A shows impaired glycosylation (**A**) Following transient transfection cell lysates (30 μg) were resolved on 10% w/v acrylamide gels and blotted with BXP-21 antibody. Transfection reagent only (PEI) served as negative control, WT His_12_–ABCG2 and His_12_–M131A serve as positive controls. His_12_–N596Q is a glycosylation defective form of ABCG2. His_12_–I573A is exclusively present at an intermediate molecular mass at 24 h and even at 48 h the majority of His_12_–I573A isoform is not the mature molecular mass. (**B**). Cell lysates were obtained 24 and 48 h post transfection, denatured and then incubated in the presence or absence of PNGaseF to remove glycosylation before western blotting. His_12_–N596Q shows no change in molecular mass upon PNGaseF treatment. By contrast, the lower molecular mass band following transfection with the His_12_–I573A isoform shows a small (estimated < 5 kDa) change in molecular mass upon PNGaseF treatment indicative of the removal of core glycosylation. WT and a representative other mutant (His_12_–M131A) are fully glycosylated at both 24 and 48 h post transfection. Data are representative of at least three independent transfections.

To enable live cell imaging of this isoform and to rule out the possibility that these results are a consequence of a transient transfection system, we generated stable cell lines expressing WT and I573A isoforms of ABCG2 with an N-terminal sfGFP extension. The presence of this tag does not affect localization of the WT protein (Figure 4A), which is expressed almost exclusively at the plasma membrane. Additionally, the drug export function of the WT isoform is unaffected by N-terminal tagging (Figure 6A) [8]. For sfGFP–I573A, the cellular fluorescence level (i.e. a measure of protein expression) is reduced compared with WT sfGFP–ABCG2 (compare intensity of Figure 4A with Figure 4B obtained using identical image acquisition and processing conditions), in agreement with data from transient expression of untagged isoforms (Figure 2). Moreover, sfGFP–I573A is retained in perinuclear and reticular intracellular compartments with very little protein at the cell surface. Co-localization of the sfGFP–I573A isoform was demonstrated with anti-calnexin antibodies confirming the ER retention (Figure 4C). ‘Rescue’ of mis-folded and mis-localized membrane proteins is of growing clinical importance [6,34,35] and has been achieved *in vitro* with either chemical chaperones (small molecules which modulate either the folding of the protein or the ER recognition of mis-folded proteins) or by reducing the rate of protein translation by lowering the cell culture temperature. We investigated both of these possibilities with the sfGFP–I573A isoform and demonstrated that neither incubation at 30°C (Figure 4D, right hand panel), nor incubation with the chaperone 4–phenylbutyrate rescued the cell surface expression of I573A (Figure 4D, left hand panel), although the latter did result in a qualitatively higher expression of sfGFP–I573A (compare Figure 4B right hand and 4D left hand panel which are obtained under identical image capture and processing settings on the same day).

**Figure 4:**
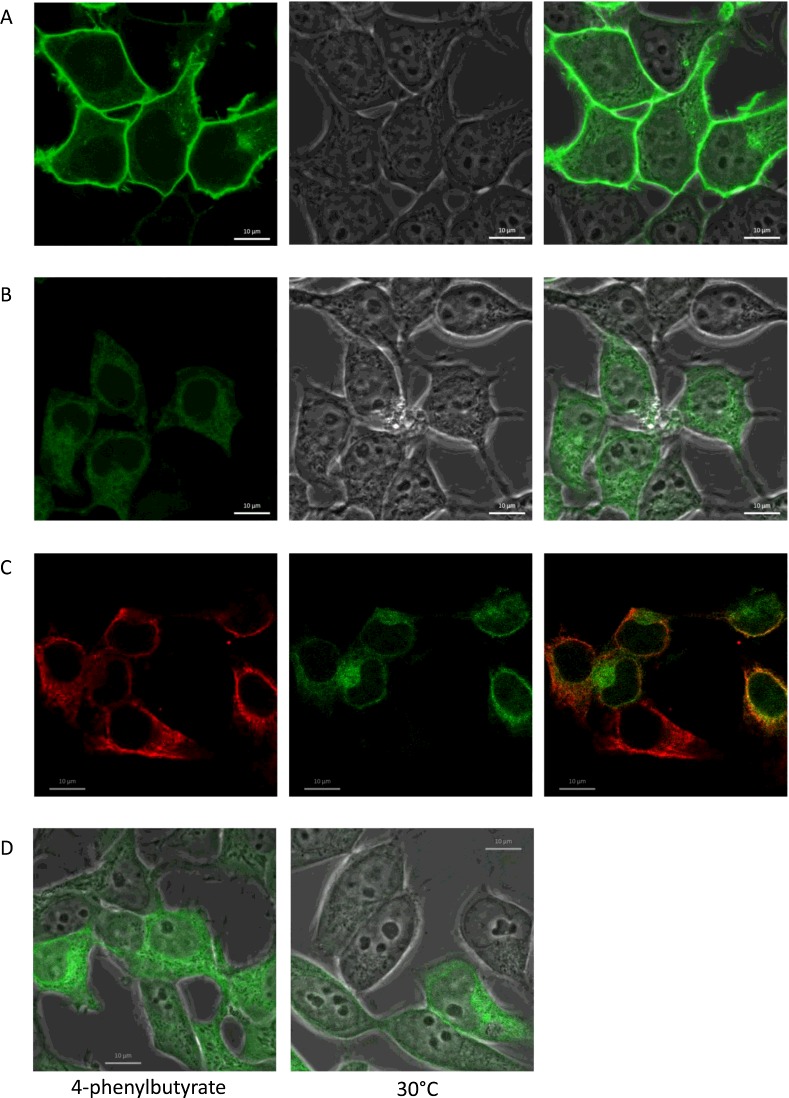
sfGFP–I573A is retained in the endoplasmic reticulum (ER) and cell surface expression is not rescued by low temperature incubation To visualize trafficking of I573 in live cells, stable cell lines expressing WT and I573A isoforms of ABCG2 with an N-terminal sfGFP tag were generated and examined by confocal imaging. The majority of WT ABCG2 is present at the cell surface (A, phase and merged image), whereas the majority of I573A is retained intracellularly (**B**). (**C**) Immunostaining of cells with anti-calnexin antibodies (red, left hand panel) shows significant co-localization of the sfGFP–I573A fluorescence signal (green, middle panel and yellow in the merged image, right hand panel) indicative of the retention of the majority of sfGFP–I573A in the ER. (**D**) Incubation of cells with the chemical chaperone 4-phenylbutyrate (5 mM) showed an increase in overall expression but no effect on localization (left hand panel). (**D**) Incubation of sfGFP-I573A expressing cells at 30°C had little effect on the localization of the I573A to the cell surface (right hand panel). Scale bar is 10 μm.

The transport function of His_12_-ABCG2 isoforms was investigated initially by flow cytometry of transiently transfected cells, taking advantage of the ability of ABCG2 to export the fluorescent drug MX and the availability of a specific ABCG2 inhibitor FTC [36,37]. Using flow cytometry, a rightward shift in the cellular fluorescence profile is expected upon incubation with FTC. The mutants could be classified into two categories, which are shown in Figure 5. Firstly, there was a group of five mutants which showed FTC-inhibited MX export comparable to WT protein (e.g. M131A, S195A, K453A, K473A, W564A; M131A data shown in Figure 3C). Secondly, there were a pair of mutant isoforms which showed little or no FTC-mediated inhibition of MX export (i.e. having high accumulation of MX even in the absence of FTC) but normal ABCG2 surface protein expression (P485A and M549A; P485A data shown in Figure 5D). These two mutants behaved in this assay in essentially the same way as the K86A and E211Q catalytically inactive mutants (result not shown).

**Figure 5:**
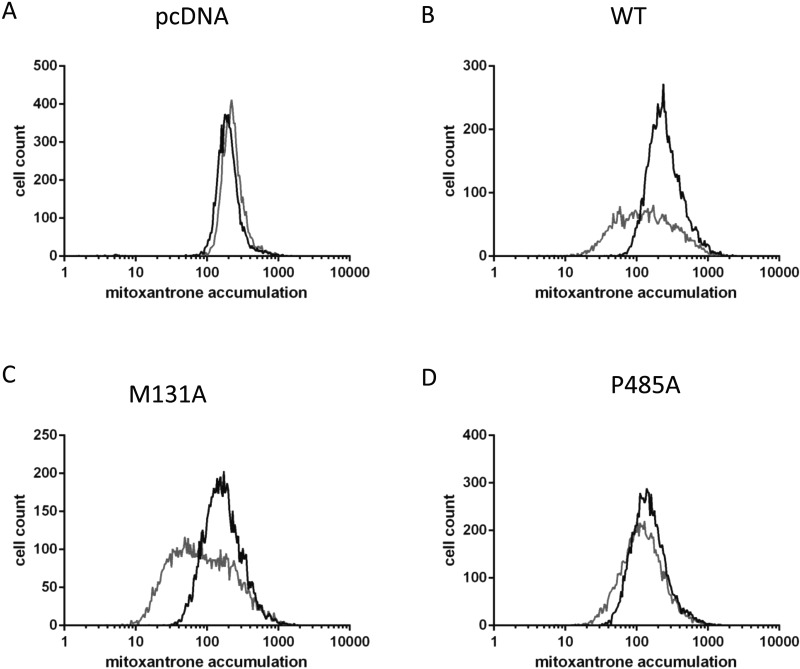
Functional analysis of His_12_–ABCG2 isoforms Functional analysis was undertaken by flow cytometry 40 h post transfection determining the cellular accumulation of MX in the absence (grey line) or presence (black line) of FTC. Data are representative of at least four independent transfections and reflect negative control data (empty vector, **A**), positive control data (WT ABCG2 expression, **B**), a mutant with normal MX transport function (M131A, **C**) and a mutant with abrogated function but normal surface expression (P485A, **D**).

To investigate further the two TMD mutations with impaired drug transport (M549A and P485A), we generated stable expressing cell lines expressing sfGFP-tagged isoforms. sfGFP-tagging was again without effect on the trafficking of the ABCG2 isoforms (Supplementary Figure S2). Transport function was examined for three substrates (MX, PhA and rhodamine 123) in a 96-well-based plate assay (Figure 6). None of the isoforms were able to transport R123, in accordance with previous data on the WT ABCG2 isoform [18]. Transport of MX differed across the mutants (Figure 6A, ANOVA, *P*<0.01). Ko143-inhibitable transport of MX was observed in the sfGFP–ABCG2–WT cell line (Figure 6A) with an approximately 5-fold increase in MX accumulation in the presence of Ko143. sfGFP–P485A retained limited MX transport function with a 1.6-fold increase in accumulation in the presence of Ko143. sfGFP–M549A and the catalytically inactive sfGFP–K86A isoform showed no Ko143-inhibited MX transport. With PhA, neither sfGFP–K86A nor sfGFP–M549A showed Ko143-inhibitable transport. In contrast, both sfGFP–ABCG2–WT and sfGFP–P485A were able to export PhA in a Ko143-inhibitable manner, with indistinguishable levels of Ko143 inhibition (Figure 6B), indicating positional specific effects on drug binding and transport.

**Figure 6:**
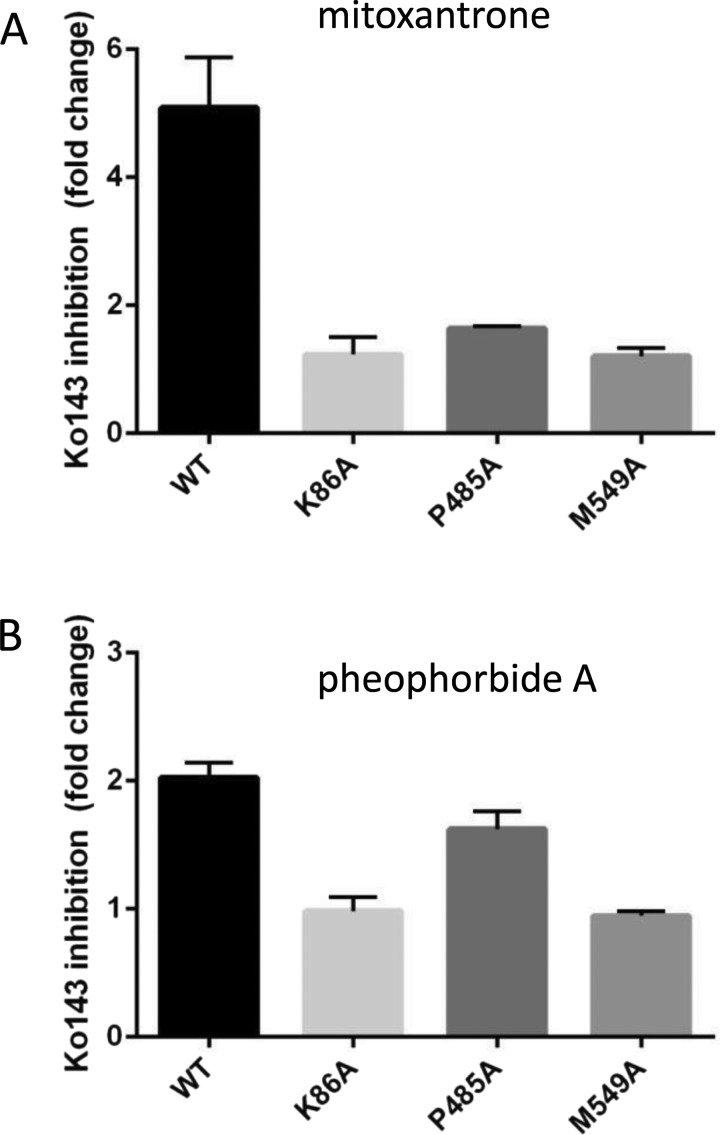
Residue Pro^485^ has a role in transport substrate specificity in ABCG2 HEK293T cells stably expressing sfGFP–ABCG2 and isoforms were analysed for transport of MTX (**A**) and PhA (**B**) using a fluorescence microplate assay. Data are expressed as the ratio of the mean fluorescence in the presence of transport substrate plus inhibitor (Ko143) compared with the mean fluorescence in the absence of the inhibitor. Data represent the mean (±S.E.M.) of at least four independent experiments. Excitation and emission wavelengths were 607 and 684 nm for MX and 390 and 680 nm for PhA respectively.

## DISCUSSION

ABCG2 is one of three human multi-drug ABC transporters and a combination of biochemical and biophysical investigations will be required to fully understand transport. Presently, our ability to interpret data on ABCG2 mutations in terms of a structural model is very limited [11]. In the present study, we have therefore utilized co-evolution analysis to identify candidate residues in ABCG2 for mutation and characterization. Two of these residues (Lys^453^ and Lys^473^) were previously mutated by Mao and colleagues [38] in a broader screen of positive charged residues in/near TM2 with similar results to those obtained in the present study. Of the eight residues we mutated, two resulted in altered drug export function (P485A and M549A) and a further residue (I573A) perturbs the trafficking and maturation of ABCG2 glycosylation. These mutants have the potential for further analysis in terms of understanding drug binding and transport, as well as trafficking of ABCG2 and its regulation on the cell surface. Intriguingly, two of the residues we have analysed (I573 and P485A) are coupled to a residue Ser^195^, which can be mutated to alanine without any apparent affect. Either this reflects that the subtle serine to alanine substitution is well tolerated at this intracellular residue or that this part of the NBD is coupled to distinct areas of the TMD through different sets of intramolecular interactions.

Residue co-evolution identifies pairs of residues whose evolution is coupled [25,26]. In studies where there is a good structural model for a protein, the importance of coupled residues is frequently examined by mutation of both the residues in the co-evolving pair. In the current paper, we have taken a slightly different approach and used co-evolution analysis to identify residues that are frequently co-evolving with other residues. We hypothesized that these residues may be involved in networks of allosteric or direct physical interactions, the disturbance of which (by mutation) will manifest in altered structure and/or function. Therefore, we mutated residues of interest to alanine as this is the simplest amino acid from a secondary structural point of view (as opposed to glycine which is likely to introduce greater local structural flexibility in the peptide backbone).

One of the eight residues we mutated in the present study (I573A) proved to have an effect on protein trafficking and maturation, an effect previously seen with a mutation of a residue located close to the intracellular side of TM1 [39]. In both transiently transfected and stably transfected cells the protein was delayed in reaching the cell surface, with a comparable delay in glycosylation. I573A is located within TM5 in the model of Mao and colleagues [30], although sequence based topological models place it within the extracellular loop between TM5 and TM6. Irrespective of its exact location, the effect of mutating I573A on targeting and glycosylation is consistent with a critical role for the TM5 and 6 region in the integrity of ABCG2 [40,41]. In contrast with some recent reports on the rescue of folding mutants by chemical ‘correctors’ or by low incubation temperatures [6,34,35], we could find not positive effect on I573A.

Two of the remaining residues we mutated (M549A and P485A) affected drug export and provide some further evidence for a role for TM3 in forming part of a drug-binding site on ABCG2. Pro^485^ is one helical turn away from position 482, the identity of which is known to be involved in substrate specificity. Our data show that mutation of Pro^485^ in TM3 results in perturbation of the drug export profile of ABCG2, with a dramatic reduction in MX transport capability but retention of PhA transport. A previous study [42] also showed P485A mutation resulted in altered substrate specificity (although the majority of MX transport was retained in that study [42]). Our resulting hypothesis from these two studies is that residue 485, like 482, contributes directly to a drug-binding pocket, although a mutation of a proline residue (which are known to cause functionally important kinks in TMs) could result in local alterations to the protein structure that would explain the effects observed. The Met^549^ residue would seem to be a poorer candidate for a direct contribution to a drug-binding site. According to the experimentally-determined topology model [30], this residue is located in the intracellular loop between TM4 and TM5, where an effect on TMD–NBD communication would be feasible if ABCG2 has intracellular coupling helices, similar to those in other ABC transporters [43]. Further analysis of purified protein preparations will be required to determine whether the lack of function of the P485A and M549A isoforms is due to impaired substrate binding (i.e. a direct effect on the drug-binding site) and/or impaired TMD–NBD communication.

## Abbreviations

ABC: ATP binding cassette
DMEM: Dulbecco's Modified Eagle Media
ER: endoplasmic reticulum
FTC: fumitremorgin C
FCS: foetal calf serum
HEK: human embryonic kidney
MX: mitoxantrone
NBD: nucleotide-binding domain
PE: phycoerythrin
PEI: polyethyleneimine
PFA: paraformaldehyde
PhA: pheophorbide A
PNGaseF: peptide–N-glycosidase F
sfGFP: superfolder GFP
TM: transmembrane helix
TMD: transmembrane domain
WT: wild type
